# Changes in Motor, Cognitive, and Behavioral Symptoms in Parkinson's Disease and Mild Cognitive Impairment During the COVID-19 Lockdown

**DOI:** 10.3389/fpsyt.2020.590134

**Published:** 2020-12-14

**Authors:** Roberta Baschi, Antonina Luca, Alessandra Nicoletti, Maria Caccamo, Calogero Edoardo Cicero, Concetta D'Agate, Lucia Di Giorgi, Giuseppe La Bianca, Tiziana Lo Castro, Mario Zappia, Roberto Monastero

**Affiliations:** ^1^Department of Biomedicine, Neuroscience and Advanced Diagnostics, University of Palermo, Palermo, Italy; ^2^Section of Neurosciences, Department of Medical and Surgical Sciences and Advanced Technologies “G.F. Ingrassia,” University of Catania, Catania, Italy

**Keywords:** COVID-19, quarantine, Parkinson's disease, cognitive impairment, behavioral symptoms, motor impairment, caregiver burden

## Abstract

**Objective:** The effects of the COVID-19 lockdown on subjects with prodromal phases of dementia are unknown. The aim of this study was to evaluate the motor, cognitive, and behavioral changes during the COVID-19 lockdown in Italy in patients with Parkinson's disease (PD) with and without mild cognitive impairment (PD-MCI and PD-NC) and in patients with MCI not associated with PD (MCInoPD).

**Methods:** A total of 34 patients with PD-NC, 31 PD-MCI, and 31 MCInoPD and their caregivers were interviewed 10 weeks after the COVID-19 lockdown in Italy, and changes in cognitive, behavioral, and motor symptoms were examined. Modified standardized scales, including the Neuropsychiatric Inventory (NPI) and the Movement Disorder Society, Unified Parkinson's Disease Rating Scale (MDS-UPDRS) Parts I and II, were administered. Multivariate logistic regression was used to evaluate associated covariates by comparing PD-NC vs. PD-MCI and MCInoPD vs. PD-MCI.

**Results:** All groups showed a worsening of cognitive (39.6%), pre-existing (37.5%), and new (26%) behavioral symptoms, and motor symptoms (35.4%) during the COVID-19 lockdown, resulting in an increased caregiver burden in 26% of cases. After multivariate analysis, PD-MCI was significantly and positively associated with the IADL lost during quarantine (OR 3.9, CI 1.61–9.58), when compared to PD-NC. In the analysis of MCInoPD vs. PD-MCI, the latter showed a statistically significant worsening of motor symptoms than MCInoPD (OR 7.4, CI 1.09–45.44). Regarding NPI items, nighttime behaviors statistically differed in MCInoPD vs. PD-MCI (16.1% vs. 48.4%, *p* = 0.007). MDS-UPDRS parts I and II revealed that PD-MCI showed a significantly higher frequency of cognitive impairment (*p* = 0.034), fatigue (*p* = 0.036), and speech (*p* = 0.013) than PD-NC. On the contrary, PD-MCI showed significantly higher frequencies in several MDS-UPDRS items compared to MCInoPD, particularly regarding pain (*p* = 0.001), turning in bed (p = 0.006), getting out of bed (*p* = 0.001), and walking and balance (*p* = 0.003).

**Conclusion:** The COVID-19 quarantine is associated with the worsening of cognitive, behavioral, and motor symptoms in subjects with PD and MCI, particularly in PD-MCI. There is a need to implement specific strategies to contain the effects of quarantine in patients with PD and cognitive impairment and their caregivers.

## Introduction

In late December 2019 an acute, severe respiratory syndrome due to coronavirus 2 (SARS-CoV-2) was reported in Wuhan, China. The illness spread rapidly worldwide, leading to the global pandemic of coronavirus disease 2019 (COVID-19). Between December 31, 2019 and July 27, 2020, there were 16,249,165 cases of COVID-19 worldwide, and 649,208 deaths have been reported. Specifically, in Europe there were 2,806,595 cases of COVID-19, with 246,118 cases in Italy resulting in 35,107 deaths^1^. In response to the growing COVID-19 pandemic in Italy, the Italian prime minister imposed a national quarantine on March 9, 2020, and a national task force of the Italian National Institute of Health was established (1). The lockdown ended on May 18, 2020, leaving far-reaching economic and social consequences. Most frequent COVID-19 clinical manifestations include cough, fever, fatigue, myalgia, gastrointestinal symptoms, and anosmia (2). The disease has also been associated with neurological abnormalities, described in up to 35% of cases (3). Reported neurological manifestations are, in decreasing order of frequency, taste/olfactory disorders (35.6%), myalgia (18.5%), headache (10.7%), stroke (8.1%), dizziness (7.9%), impaired consciousness (7.8%), and seizure (1.5%) (4).

Neurodegenerative disorders, including Alzheimer's disease (AD) and Parkinson's disease (PD), are very prevalent diseases in the elderly, constituting some of the greatest future medical challenges, due to aging populations. These subjects are frail individuals with specific cognitive, motor, and behavioral symptoms with inherent problems of adaptation to changes and environmental stressors (5, 6). Furthermore, neurodegenerative diseases have been associated with an increased burden for the caregiver, leading to behavioral disturbances, particularly depression (7).

The clinical presentation of COVID-19 in dementia is rather atypical. Its onset is often characterized by delirium, particularly of the hypoactive form, and worsening disability (8). Furthermore, a pre-existing diagnosis of dementia is an independent risk factor for COVID-19 hospitalization and related mortality in the UK Biobank Community Cohort (9). The effect of COVID-19 quarantine on subjects with dementia and its preclinical phase (i.e., Mild Cognitive Impairment (MCI) (10) has been poorly investigated. In a French report conducted in 38 AD patients, approximately 25% showed new symptoms or a worsening of pre-existing neuropsychiatric symptoms (NPS) during the lockdown period (11). Others reported that the most affected NPS symptoms during confinement in MCI and AD were apathy, anxiety, agitation, and wandering (12).

The authors of the present study know of no PD studies specifically evaluating the effect of COVID-19 on patients with PD and cognitive impairment. It was reported in recent research that COVID-19 significantly worsened motor and non-motor symptoms in PD, although cognitive functioning was marginally involved. However, these authors did not include subjects with PD with mild cognitive impairment (PD-MCI), nor did they perform a baseline neuropsychological evaluation (13).

Overall, there is a paucity of knowledge relating to the cognitive, motor, and behavioral symptoms in patients with PD during the COVID-19 quarantine, particularly in those with PD-MCI. Furthermore, few data have been described for MCI, the intermediate state between normal aging and AD. Therefore, there is a need for research to assess the impact of the COVID-19 lockdown on the natural course of the preclinical phases of dementia, which are associated with AD and PD. To answer this question, the aim of the present study was to evaluate cognitive, behavioral, and motor changes in patients with PD and MCI during the COVID-19 lockdown in Italy. The specific research aims of the study were 2-fold: to evaluate the impact of cognitive impairment during the COVID-19 lockdown in subjects with PD, for this purpose, collected data were compared in PD patients with and without MCI; and to evaluate the impact of motor impairment in subjects with MCI, collected data from PD-MCI patients vs. MCI not associated with PD (MCInoPD) were compared.

## Materials and Methods

### Study Population

A cross-sectional study was carried out that included 96 patients: 34 with PD with normal cognition (PD-NC), 31 affected by PD-MCI, and 31 with MCInoPD. Patients who underwent a comprehensive motor, clinical, and cognitive assessment (i.e., baseline assessment) in the 2 months preceding the COVID-19 lockdown in Italy (from March 9, 2020 to May 18, 2020) (14, 15) were enrolled. Subjects attended the “Parkinson's disease and Movement Disorders Center” and the “Memory Center” of the Neurologic Unit of the “Policlinico Vittorio Emanuele” in Catania and the Memory and Parkinson's Disease Center of the “Policlinico Paolo Giaccone” in Palermo, all of which belong to the PArkinson's disease Cognitive impairment Study [PACOS] and to the PREclinical Cognitive Impairment Study in the Elderly [PRECISE].

#### The PACOS Baseline Assessment

The PACOS study is a prospective cohort study, aimed at evaluating frequency, clinical features, and biomarkers associated with MCI in a large hospital-based sample of PD patients, whose details have been previously described (16, 17). All patients underwent a standard neurological examination performed by neurologists experienced in movement disorders, including the Unified Parkinson Disease Rating Scale—Motor Evaluation (UPDRS-ME) (18) and the Hoehn and Yahr scale (19). The overall burden of dopaminergic drugs was evaluated with the Levodopa Equivalent Daily Dosage (LED) (20, 21). Patients were classified according to cognitive performance as PD-NC or PD-MCI. Functional independence was assessed with the Basic and the Instrumental Activities of Daily Living (BADL and IADL, respectively) (22, 23), and scored as the number of items lost for each scale. Inclusion criteria for this study were a diagnosis of PD according to the Brain Bank criteria (24); a diagnosis of PD-MCI according to MDS level II criteria (25); and mild-moderate PD (e.g., Hoehn and Yahr Stage I–III). The exclusion criteria were the presence of significant depression, excluded using the Hamilton Depression Rating Scale, considering cut-off scores as suggested by the MDS (26) and a diagnosis of dementia in PD (PDD), according to the MDS task force criteria for PDD (27).

#### The PRECISE Baseline Assessment

The PRECISE is a prospective, cohort study aimed at evaluating clinical, cognitive, behavioral, and biomarkers associated with Subjective Cognitive Decline and MCI in a large hospital-based sample of the elderly, which began recently and is still ongoing. Inclusion criteria were a diagnosis of MCInoPD according to modified Petersen's criteria (28) as follows: (1) self and/or informant report of cognitive complaint; and (2) objective cognitive impairment (all subjects underwent a neuropsychological assessment including the Mini Mental State Examination (MMSE) (29) as a test of global cognition). Subsequently, a complete neuropsychological battery including two tests for each cognitive domain (e.g., episodic memory, selective and divided attention, executive functioning, language, and visuospatial functioning) was administered. Details regarding administration procedures and Italian normative data for score adjustment, based on age and education, as well as normality cut-off scores (≥95% of the lower tolerance limit of the normal population distribution), were available for each test battery (30, 31); (3) preserved independence in functional abilities [e.g., preserved number of BADL (22) and with no or minimal impairment regarding IADL lost (23)]; and (4) cognitive deterioration, representing a decline from a previously higher ability level [global Clinical Dementia Rating scale score of 0.5 (32)]. Current depressive symptoms were assessed by the 15-item version of the Geriatric Depression Scale at the recommended cut-off score of 5 (33). Exclusion criteria were (1) a diagnosis of dementia according to the DSM-IV-TR criteria (34) and (2) vascular cognitive impairment, according to clinical history of stroke and a routine 1.5T MRI scan (Signa HDxt; GE Medical System, Milwaukee, WI, USA) to exclude relevant vascular lesions, which would affect cognition, as described previously (35).

All participants provided written fully informed consent prior to entering the studies, which were approved by the local Ethics Committee (PACOS approval number 3/2018; PRECISE approval number 01/2019) and were in accordance with the Declaration of Helsinki.

### Caregivers' and Patients' Interview Related to the COVID-19 Lockdown Period

Telephone interviews were carried out with the patients and their caregivers immediately after the end of the COVID-19 lockdown in Italy (May 20–30, 2020). All participants provided initially oral and then written informed consent prior to entering the COVID extension study (Ethics Committee approval number 5/2020).

Due to the different assessment modalities (face-to-face examination at baseline vs. telephone interview after quarantine), the telephone interview included data obtained from caregivers using a semi-structured questionnaire as well as modified, standardized scales, which were administered to the caregivers and the patients, as follows:

- *Caregiver questionnaire*: An *ad hoc* questionnaire was administered, including specific dichotomic questions (e.g., presence vs. absence) regarding patients' modifications in cognitive, behavioral, and motor symptoms during the quarantine. Specifically, caregivers were asked about cognitive changes through questions assessing the new onset/worsening of memory deficits, temporospatial disorientation, word-finding difficulty, confusion, and topographical disorientation. Behavioral symptoms were assessed using the entry question for each Neuropsychiatric Inventory (NPI) domain (36) by evaluating the worsening of pre-existing and the new onset of neuropsychiatric symptoms (NPS) occurring during the lockdown period. Referring to new onset/worsening of motor symptoms, questions related to motor slowing, tremor, difficulty in getting out of the bed, and rising from a chair were administered. At the end of this section, the caregiver was asked to indicate whether the disease had worsened during quarantine and whether its burden had increased during the lockdown.
- *Standardized scales*: Included the evaluation of cognitive, behavioral, and motor modifications with respect to the commencement of the lockdown period. Changes in global cognition were evaluated with the Italian telephone version of the MMSE (Itel-MMSE) (37). Behavioral modifications were carried out with the NPI, a fully structured caregiver interview, which measures 12 behavioral symptoms (36). For the purpose of the present research, only the presence–absence of each symptom was collected by evaluating the worsening of pre-existing and the new onset of NPS. Non-motor and motor changes were evaluated with the MDS-UPDRS Parts I and II, which scored non-motor and motor aspects of daily living experiences, respectively. For the present research, original questions were slightly modified, using a dichotomous variable with 0 = symptom absent or stable and 1 = symptom worsened and/or of new onset. Lastly, the number of IADL lost after quarantine was evaluated (25).

### Statistical Analysis

The data were analyzed using IBM SPSS Statistics for Windows, Version 20.0 (IBM Corp., Armonk, NY, USA). Data cleaning was performed prior to data analysis by considering range and consistency checks. Quantitative variables were described using mean and standard deviation, while qualitative variables were expressed as number and percentage. The demographic and clinical variables among groups were evaluated with one-way analysis of variance (ANOVA) with *post hoc* Scheffe's test for multiple comparisons and chi-square analysis followed by Fisher's exact test, as required. For all analyses the significance level was set at 0.05.

In order to evaluate variables associated with PD-MCI (outcome variable), an unconditional logistic regression analysis was performed for each study variable. Two different analyses were performed: (1) to test the impact of cognitive impairment during the COVID-19 lockdown period in subjects with PD, PD-NC was compared to PD-MCI; and (2) to evaluate the impact of motor impairment in subjects with MCInoPD, the latter was compared to PD-MCI. Regarding logistic regression analysis, the odds ratios (OR) with 95% confidence intervals (CI) were calculated. Parameters associated with the outcome at the univariate analysis with *p*-value ≤0.05 were included in the final multivariate analysis, which was further adjusted for age, gender, and education, considered a priori confounders.

## Results

### Baseline Characteristics of PD-NC, PD-MCI, and MCInoPD

A total of 96 patients, 34 with PD-NC, 31 affected by PD-MCI, and 31 with MCInoPD, were enrolled in the present study. With regard to demographic characteristics, the groups did not differ by age (*p* = 0.238), gender (*p* = 0.242), and education (*p* = 0.724) (see Table 1). ANOVA revealed a significant disease-duration effect between groups (*p* < 0.0001), and after Scheffe's *post hoc* analysis, PD-MCI showed a significantly longer disease duration than MCInoPD (*p* < 0.0001).

**Table 1:** Baseline characteristics of PD-NC, PD-MCI, and MCInoPD.

	**Total** **(*n* = 96)**	**PD-NC** **(*n* = 34)**	**PD-MCI** **(*n* = 31)**	**MCInoPD** **(*n* = 31)**	***p*-value**	***post hoc*** ***p*-value** ***PD-NC vs. PD-MCI***	***post hoc*** ***p*-value** ***PD-MCI vs. MCInoPD***
***Patients***							
Age, years	67.3 ± 11.2	65.4 ± 9.6	66.7 ± 14.7	70.0 ± 8.3	0.238	0.664	0.281
Gender male (*n*, %)	58 (60.4)	23 (67.6)	20 (64.5)	15 (48.4)	0.242	0.790	0.200
Education, years	9.9 ± 4.2	10.1 ± 4.2	10.2 ± 4.1	9.4 ± 4.2	0.724	0.944	0.469
Disease duration, years	4.6 ± 3.6	6.2 ± 3.8	6.5 ± 2.8	1.7 ± 1.1	<0.0001	0.769	<0.0001
MMSE	26.4 ± 2.7	27.9 ± 1.8	26.1 ± 2.0	25.0 ± 3.2	<0.0001	<0.0001	0.099
LED (mg/day)	–	537.3 ± 407.5	502.3 ± 319.0	–		0.704	–
Hoehn and Yahr	–	2.2 ± 0.5	2.4 ± 0.5	–		0.192	–
Number of BADL lost	0.3 ± 0.6	0.06 ± 0.2	0.8 ± 0.9	0.1 ± 0.3	<0.0001	<0.0001	<0.0001
Number of IADL lost	0.7 ± 1.2	0.1 ± 0.4	1.3 ± 1.4	0.7 ± 1.1	<0.0001	<0.0001	0.065
***Caregivers***							
Age, years	61.4 ± 12.6	61.0 ± 12.1	62.6 ± 12.5	60.5 ± 13.5	0.800	0.613	0.535
Gender male (*n*, %)	28 (29.2)	11 (32.4)	9 (29)	8 (25.8)	0.845	0.772	0.776
Education, years	10.2 ± 4.0	10.5 ± 3.8	9.6 ± 4.1	10.5 ± 4.2	0.604	0.363	0.411

*Data are expressed as mean ± standard deviation and number and percentage. PD-NC, Parkinson's disease with normal cognition; PD-MCI, Parkinson's disease with mild cognitive impairment; MCInoPD, mild cognitive impairment not associated with Parkinson's disease; MMSE, Mini Mental State Examination; LED, Levodopa Equivalent Daily Dosage; BADL, Basic Activities of Daily Living; IADL, Instrumental Activities of Daily Living*.

Concerning overall cognition and disability, ANOVA showed significant differences in MMSE score (*p* < 0.0001), the number of BADL (*p* < 0.0001), and IADL (*p* < 0.0001) lost within groups. A *post hoc* analysis revealed that PD-MCI had a significant lower MMSE performance (*p* < 0.0001) and a higher number of IADL lost (*p* < 0.0001) than PD-NC, while, as expected, the latter group showed a significantly better performance in BADL than both PD-MCI and MCInoPD (*p* < 0.0001 for both comparisons).

With respect to motor parameters, there was no significant difference in LED and the Hoehn and Yahr score between PD-NC and PD-MCI (*p* = 0.704 and *p* = 0.192, respectively). Lastly, with regard to caregivers' features, no significant differences in ANOVA were observed relating to age (*p* = 0.800), gender (*p* = 0.845), and education (*p* = 0.604) between groups.

### Influence of COVID-19 Quarantine on Cognitive, Behavioral, and Motor Symptoms in PD-NC, PD-MCI, and MCInoPD

ANOVA revealed significant differences concerning global cognition after quarantine in the Itel-MMSE score within groups (*p* = 0.008), although there were no significant differences regarding MMSE scores in pair comparisons. In addition, the number of IADL lost after quarantine significantly differed in ANOVA between groups (*p* ≤ 0.0001), with PD-MCI showing the highest number of IADL lost, compared with PD-NC (*p* ≤ 0.0001), and MCInoPD (*p* = 0.047) (see Table 2).

**Table 2:** Influence of COVID-19 quarantine on cognitive, behavioral, and motor symptoms in PD-NC, PD-MCI, and MCInoPD.

	**PD-NC** **(*n* = 34)**	**PD-MCI** **(*n* = 31)**	**MCInoPD** **(*n* = 31)**	***p*-value**	***post hoc*** ***p*-value** ***PD-NC vs. PD-MCI***	***post hoc*** ***p*-value** ***PD-MCI vs. MCInoPD***
Itel-MMSE after quarantine	24.3 ± 2.8	22.9 ± 2.4	22.1 ± 3.3	0.008	0.149	0.532
Number of IADL lost after quarantine	0.1 ± 0.5	1.6 ± 1.8	0.8 ± 1.2	<0.0001	<0.0001	0.047
Worsening of cognition^*^	10 (30.3)	14 (45.2)	13 (41.9)	0.544	0.289	0.798
Worsening of pre-existent NPS^*^	11 (32.4)	13 (41.9)	12 (38.7)	0.718	0.424	0.796
New onset of NPS^*^	5 (14.7)	13 (41.9)	7 (22.6)	0.038	0.014	0.103
Worsening of motor symptoms^*^	18 (52.9)	14 (45.2)	2 (6.4)	<0.0001	0.531	<0.0001
Disease acceleration^*^	8 (23.5)	3 (9.7)	5 (16.3)	0.325	0.137	0.449
Increased caregiver burden^*^	11 (32.3)	11 (35.5)	3 (9.7)	0.040	0.790	0.015

*Data are expressed as mean ± standard deviation and number and percentage. PD-NC, Parkinson's disease with normal cognition; PD-MCI, Parkinson's disease with mild cognitive impairment; MCInoPD, mild cognitive impairment not associated with Parkinson's disease; Itel-MMSE, Italian telephone Mini Mental State Examination; IADL, Instrumental Activities of Daily Living; NPS, neuropsychiatric symptoms*.

* *data obtained from caregiver interview*.

Based on caregivers' reports, no significant differences between groups in ANOVA were observed in worsening of cognition (*p* = 0.544), worsening of pre-existent NPS (*p* = 0.718), and disease acceleration (*p* = 0.325). On the contrary, ANOVA revealed a significant difference in the new onset of NPS within groups (*p* = 0.038), with PD-MCI showing a significantly higher new onset of NPS than PD-NC (*p* = 0.014) in pair comparison. In addition, ANOVA revealed significant differences between groups in the worsening of motor symptoms (*p* ≤ 0.0001) and increased caregiver burden (p = 0.040). Specifically, the latter differences proved to be significant at pair comparison for PD-MCI vs. MCInoPD, with the former group showing a significantly higher burden than the latter regarding worsening of motor symptoms (*p* ≤ 0.0001) and increased caregiver burden (*p* = 0.015). Of interest, a significant difference in pair comparison concerning worsening of motor symptoms (*p* ≤ 0.0001) and increased caregiver burden (*p* = 0.026) was also observed when comparing PD vs. MCInoPD (data not shown).

### Univariate and Multivariate Analysis of Cognitive, Behavioral, and Motor Changes After COVID-19 Quarantine in PD-NC vs. PD-MCI and MCInoPD vs. PD-MCI

#### PD-NC vs. PD-MCI

First, PD-NC and PD-MCI were compared using logistic regression analysis to evaluate the effect of quarantine on global cognition, disability, and behavioral and motor changes between groups. As expected, the Itel-MMSE after quarantine was significantly higher in subjects with PD-NC than with PD-MCI (OR 0.8, CI 0.66–0.99). Furthermore, the number of IADL lost after quarantine was positively associated with PD-MCI (OR 3.6, CI 1.53–8.41). After multivariate analysis and considering age, gender, and education as a priori confounders, the IADL lost after quarantine (OR 3.9, CI 1.61–9.58) and the Itel-MMSE (OR 0.7, CI 0.55–0.97) were still statistically significant. On the contrary, with respect to variables collected through caregiver reports after quarantine (e.g., changes in NPS, motor and cognitive status, disease acceleration, and caregiver burden), all these comparisons were not significant at univariate analysis between PD-NC and PD-MCI.

#### MCInoPD vs. PD-MCI

As regards the comparison between MCInoPD and PD-MCI, univariate analysis showed that the number of IADL lost after quarantine was positively associated with PD-MCI (OR 1.4, CI 1.0–2.03), while no significant difference between groups in the Itel-MMSE was observed. However, the association between PD-MCI and number of IADL lost after quarantine was not confirmed after multivariate analysis (see Table 3). Regarding caregivers' report, univariate analysis showed that worsening of motor symptoms (OR 11.9, CI 2.41–59.03) and increased caregiver burden (OR 5.1, CI 1.27–20.81) were positively associated with PD-MCI. However, after multivariate analysis, only the association between worsening of motor symptoms and PD-MCI was confirmed (OR 7.4, CI 1.09–45.44).

**Table 3:** Univariate and multivariate analysis of cognitive, behavioral, and motor changes after Covid-19 quarantine in PD-NC vs. PD-MCI and MCInoPD vs. PD-MCI.

	**PD-NC vs. PD-MCI**						**MCInoPD vs. PD-MCI**					
	**Univariate analysis**			**Multivariate analysis**			**Univariate analysis**			**Multivariate analysis**		
	**OR**	**95% CI**	***p*-value**	**OR**	**95% CI**	***p*-value**	**OR**	**95% CI**	***p*-value**	**OR**	**95% CI**	***p*-value**
Age, years^#^	1.0	0.97–1.10	0.660	1.0	0.92–1.07	0.921	1.0	0.93–1.02	0.296	1.0	0.91–1.07	0.743
Gender, male^#^	0.9	0.31–2.43	0.790	1.9	0.43–8.18	0.399	1.9	0.70–5.37	0.202	1.7	0.47–6.28	0.413
Education, years^#^	1.0	0.89–1.13	0.943	1.1	0.94–1.39	0.193	1.0	0.92–1.18	0.463	1.0	0.88–1.20	0.704
Itel-MMSE after quarantine	0.8	0.66–0.99	0.041	0.7	0.55–0.97	0.031	1.1	0.93–1.32	0.270			
IADL lost after quarantine	3.6	1.53–8.41	0.003	3.9	1.61–9.58	0.003	1.4	1.0–2.03	0.048	1.1	0.64–1.76	0.702
Worsening of cognition^*^	1.7	0.62–4.72	0.291				1.1	0.42–3.11	0.798			
Worsening of pre-existent NPS^*^	1.5	0.54–4.15	0.425				1.1	0.41–3.16	0.796			
New onset of NPS^*^	4.2	1.28–13.73	0.18				2.5	0.82–7.47	0.107			
Worsening of motor symptoms^*^	0.7	0.28–1.94	0.531				11.9	2.41–59.03	0.002	7.4	1.09–45.44	0.040
Disease acceleration^*^	0.3	0.08–1.45	0.148				0.6	0.12–2.57	0.453			
Increased caregiver burden^*^	1.2	0.41–3.21	0.790				5.1	1.27–20.81	0.022	2.1	0.36–12.52	0.408

*PD-NC, Parkinson's disease with normal cognition; PD-MCI, Parkinson's Disease with mild cognitive impairment; MCInoPD, mild cognitive impairment not associated with Parkinson's disease; OR, odds ratio; CI, confidence interval; Itel-MMSE, Italian telephone Mini Mental State Examination; IADL, Instrumental Activities of Daily Living; NPS, neuropsychiatric symptoms*.

# *Considered a priori confounders*;

* *data obtained from caregiver interview*.

Of interest, a diagnosis of PD was also positively associated with worsening of motor symptoms (OR 16.3, CI 3.35–79.46) and increased caregiver burden (OR 5.1, CI 1.27–20.81) at univariate analysis in comparison with MCInoPD subjects. This result was confirmed after multivariate analysis (worsening of motor symptoms: OR 20.4, CI 3.66–113.98; increased caregiver burden: OR 4.9, CI 1.06–22.83) (data not shown).

### NPI and MDS-UPDRS Changes in PD-NC, PD-MCI, and MCInoPD During Quarantine

**Figure 1:**
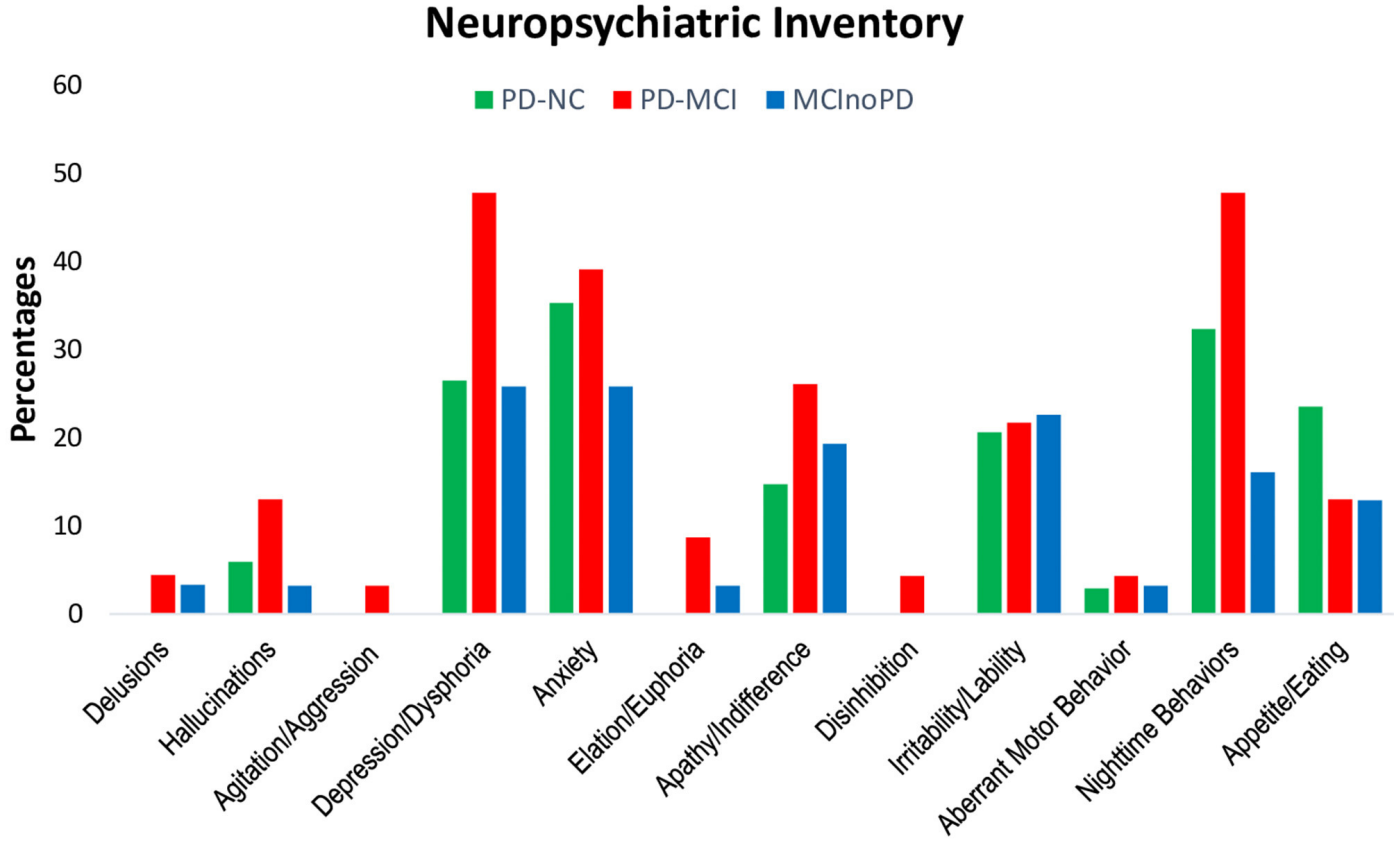
Changes in NPI in PD-NC, PD-MCI and MCInoPD. NPI, Neuropsychiatric Inventory; PD-NC, Parkinson's disease with Normal Cognition; PD-MCI, Parkinson's disease with Mild Cognitive Impairment; MCInoPD, Mild Cognitive Impairment not associated with Parkinson's Disease.

First, changes during the lockdown period of NPS in NPI were evaluated (see Supplementary Tables 1a, 2a; Figure 1). Except for appetite/eating disturbances, subjects with PD-MCI showed higher frequencies in all the NPI symptoms, when compared to PD-NC. However, a significant, borderline trend was found only for depression (*p* = 0.067), euphoria (*p* = 0.063), and aberrant motor behavior (*p* = 0.067). Similarly, PD-MCI showed higher frequencies of NPS in all NPI domains when compared to MCInoPD. Significant results were found for nighttime behaviors (*p* = 0.007), with borderline significant results for depression (*p* = 0.066) and aberrant motor behavior (*p* = 0.086).

**Figure 2:**
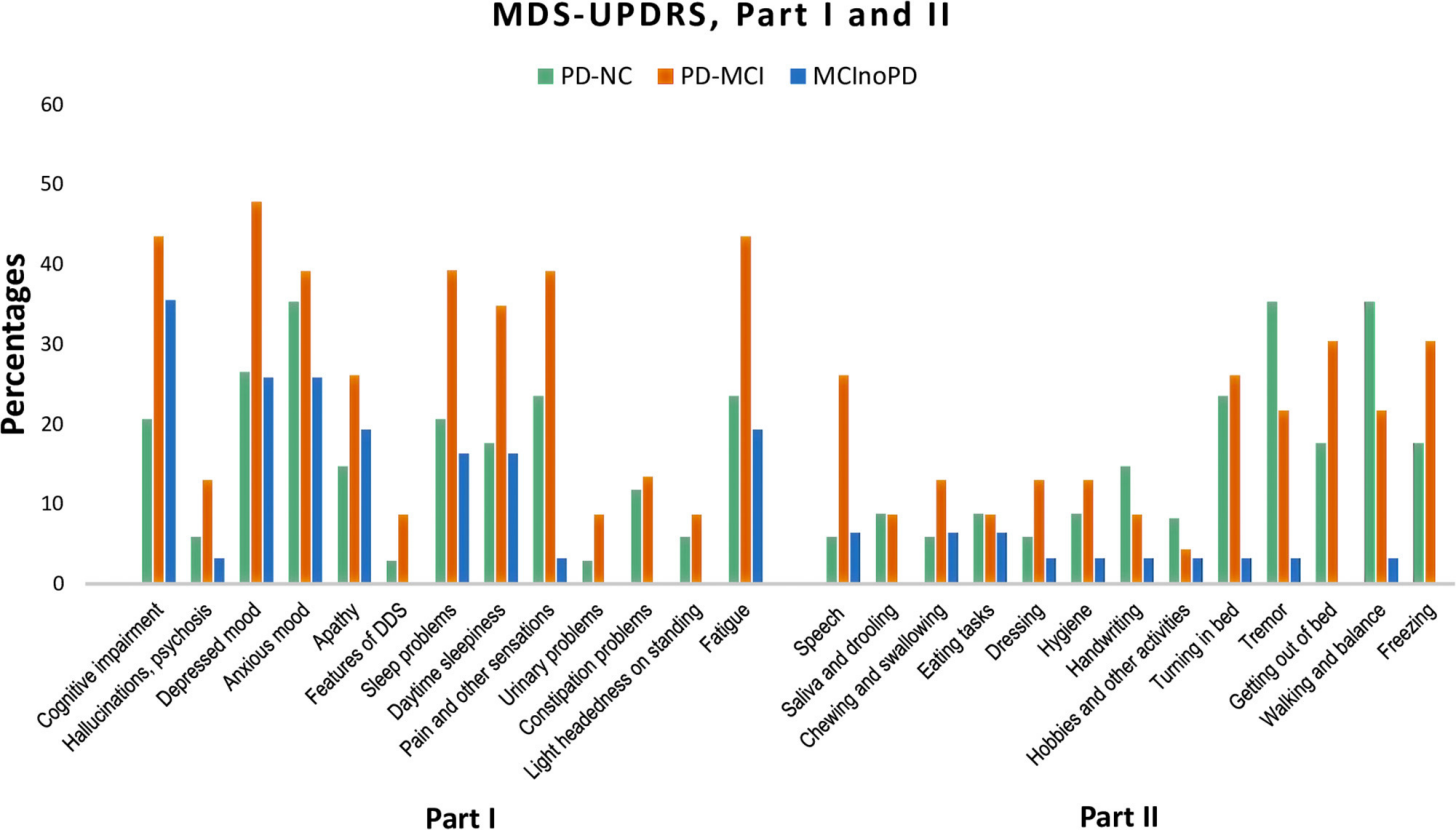
Changes in MDS-UPDRS Part I and Part II in PD-NC, PD-MCI and MCInoPD. MDS-UPDRS, Movement Disorder Society Unified Parkinson's Disease Rating Scale; PD-NC, Parkinson's disease with Normal Cognition; PD-MCI, Parkinson's Disease with Mild Cognitive Impairment; MCInoPD, Mild Cognitive Impairment not associated with Parkinson's Disease. DDS, Dopamine Dysregulation Syndrome.

Changes during the quarantine period in MDS-UPDRS Parts I and II were also evaluated (see Supplementary Tables 1b, 2b; Figure 2). Concerning MDS-UPDRS Part I in PD-NC vs. PD-MCI, the latter showed significant higher frequencies in cognitive impairment (*p* = 0.034) and fatigue (*p* = 0.036) than the former, with borderline significant differences for depressed mood (*p* = 0.067) and sleep problems (*p* = 0.063). With respect to MDS-UPDRS Part II, patients with PD-MCI showed significant higher frequencies than PD-NC in speech (*p* = 0.013). Subsequently, MDS-UPDRS items were compared in MCInoPD vs. PD-MCI. As regards MDS-UPDRS Part I, PD-MCI showed significant higher frequencies for sleep problems (*p* = 0.025), pain and other sensations (*p* = 0.001), urinary problems (*p* = 0.039), constipation problems (*p* = 0.039) and fatigue (*p* = 0.016), with borderline significant differences for depressed mood (*p* = 0.066), daytime sleepiness (*p* = 0.082), and light-headedness on standing (*p* = 0.076). Regarding MDS-UPDRS Part II, PD-MCI showed significant higher frequencies in speech (*p* = 0.020), saliva and drooling (*p* = 0.039), turning in bed (*p* = 0.006), tremor (*p* = 0.012), getting out of bed (*p* = 0.001), and walking and balance (*p* = 0.003), with borderline significance values for dressing (*p* = 0.086) and hygiene (*p* = 0.086).

## Discussion

The present research evaluated the impact of 10 weeks of lockdown in PD patients with and without MCI and in subjects with MCI not associated with PD during the COVID-19 epidemic in Italy. COVID-19 quarantine led to a worsening of cognitive, behavioral, and motor symptoms in subjects with PD and MCI. According to the caregivers' reports, social distancing and isolation due to lockdown led to a relevant worsening of cognition in nearly 40% of patients, worsening of NPS in 37.5%, new onset of NPS in 26%, and worsening of motor functioning in approximately 35% of patients. Consequently, the caregiver's burden during lockdown increased in over 25% of cases.

Concerning the baseline characteristics of the study groups, subjects with PD-MCI showed a significant longer disease duration than MCInoPD. Regarding global cognition, the latter group performed worse at baseline MMSE than both PD-NC and PD-MCI, but this result was significant only when comparing MCInoPD vs. PD-NC. Similarly, a significant difference within groups was found for BADL and IADL, with PD-MCI showing the highest number of activities lost, when compared to PD-NC and MCInoPD. With reference to the caregivers' characteristics, no differences were found within groups. An examination of the parameters collected after the COVID-19 quarantine revealed a significant difference regarding the Itel-MMSE within groups, although groups did not differ at pair comparison. Contrarily, PD-MCI lost a significantly higher number of IADL during the lockdown period comparing PD-NC and MCInoPD. An examination of variables collected via caregivers' reports revealed a significant difference for new onset NPS, and this occurred with a higher frequency in PD-MCI vs. PD-NC. Furthermore, worsening of motor symptoms and increased caregiver burden significantly increased during the lockdown period in both PD groups, compared to MCInoPD.

First, the effect of cognitive impairment in PD during the COVID-19 lockdown period was examined by comparing PD-NC vs. PD-MCI patients. A multivariate logistic regression analysis demonstrated that the number of IADL lost during the quarantine period was significantly and positively associated with PD-MCI. Furthermore, and as expected, PD-NC patients showed significant higher Itel-MMSE scores than PD-MCI. The two PD groups did not significantly differ in the worsening of their cognitive, behavioral, and motor symptoms, disease acceleration, and increased caregiver burden. Concerning NPI and MDS-UPDRS Parts I and II performance after the lockdown period, the effect of quarantine in subjects with PD was mainly associated with cognitive impairment. Indeed, PD-NC vs. PD-MCI did not differ with respect to motor symptoms, while the latter showed significantly higher frequencies of cognitive impairment and speech disturbances than the former. The authors of the present study know of only one study that examined the effect of COVID-19 on patients with PD, comparing motor and non-motor symptoms in subjects with and without the infection (13). Those authors found that clinical symptoms significantly worsened in the COVID-19 group, although cognitive functioning was marginally involved. However, the authors of that study did not specifically evaluate subjects with PD-MCI and, moreover, they did not assess the effects of COVID quarantine in patients with PD.

Subsequently, the effect of motor impairment in MCI during the COVID-19 lockdown was assessed comparing MCInoPD vs. PD-MCI patients. Multivariate logistic regression analysis demonstrated that PD-MCI was significantly and positively associated with the worsening of motor symptoms, with a subsequent increased caregiver burden compared to MCInoPD, and this result was also confirmed when comparing PD-NC vs. MCInoPD. The latter result suggests that social isolation and a reduction in cognitive stimulation and physical activity during the COVID-19 lockdown period differentially impact subjects with neurodegenerative diseases. Specifically, PD patients seem to be more susceptible than MCInoPD, regardless of the presence/absence of a concomitant cognitive impairment. Concerning NPI and MDS-UPDRS performance after confinement, the present data suggest that PD-MCI showed a higher frequency of motor (e.g., turning in bed, tremor, getting out of bed, and walking/balance) and non-motor symptoms (sleep problems and nighttime behaviors, pain, urinary and constipation problems, and fatigue) than MCInoPD. The authors of the present study are aware of only one study to date that describes the effect of the COVID-19 quarantine period on subjects with MCI (12). In this Spanish report, the authors found that the most affected NPS symptoms during confinement in 20 MCI subjects were apathy and anxiety. These data confirm the results of the present study, which showed that MCInoPD patients overall had a significant worsening in NPS during COVID-19 confinement. However, in the Lara et al. study (12), the authors did not include subjects with PD, thus a comparison of this study with data from the present research is not entirely feasible.

Overall, the results of the present research suggest that the COVID-19 quarantine in subjects with MCI has a greater impact on PD-specific symptoms, rather than cognitive. This is probably due to the obligatory increase in sedentary lifestyle due to the COVID-19 confinement in the elderly with motor impairment, such as those with PD, leading to a greater deterioration in cognitive and behavioral functioning compared to those subjects without motor impairment. Indeed, it is well-known that physical inactivity is a risk factor for cognitive impairment and depression, also exacerbating various non-motor symptoms, including insomnia and constipation (38). This issue was tested recently by a double-blind randomized controlled trial, which found that a multidomain intervention (including regular exercise) could improve or maintain cognitive functioning in at-risk elderly individuals from the general population (39).

In the present research, a comprehensive assessment aimed at evaluating the effects of quarantine in Italian patients with PD and MCI was conducted. Caregivers and patients completed an *ad hoc*, semi-structured questionnaire, in addition to standardized scales to evaluate the effect of social distancing and isolation during the lockdown. To the best of our knowledge, this is the first study that has investigated the effects of quarantine in patients with PD-MCI, comparing the latter to those with MCInoPD.

Nevertheless, some limitations of the present study should be pointed out. First, the relatively small sample size of the groups might increase the likelihood of spurious associations and a lack of significance (e.g., the results approached significance for many NPI and MDS-UPDRS items). Second, although analyses were adjusted for potential confounders, residual confounding (e.g., medical comorbidity, the use of psychotropic drugs) cannot be excluded. Third, MCInoPD was diagnosed according to clinical criteria (28), with some inevitable uncertainty about diagnostic accuracy. MCI is a condition with multiple sources of heterogeneity, including clinical presentation, etiology, and prognosis (10). However, a comprehensive clinical protocol for MCInoPD was used and diagnoses were supported by brain imaging. Fourth, due to the cross-sectional design of the study, it is unclear whether the observed clinical worsening represents a transient or a persistent phenomenon. For this reason, a follow-up evaluation of patients has already been envisaged. Finally, caregiver rating bias was also reported in subjects with MCInoPD, and it may well be associated with the caregiver burden (40). Accordingly, data based on caregiver ratings should be interpreted with caution due to the increased caregiver burden described in the present research.

In conclusion, results of the present research show that the COVID-19 related-quarantine has exacerbated cognitive, behavioral, and motor symptoms in subjects with PD, particularly in PD-MCI. The Italian National Health Care System needs to plan specific health strategies to guarantee appropriate care in subjects with cognitive impairment and their caregivers. To this end, telemedicine and digital technology devices would be of particular assistance in remote monitoring and care of subjects with cognitive impairment during confinement due to the COVID-19 pandemic.

## Data Availability Statement

Anonymized raw data are available by the authors if required.

## Ethics Statement

The studies involving human participants were reviewed and approved by Ethics Committee Palermo 1: approval number: 5/2020. The patients/participants provided their written informed consent to participate in this study.

## Author Contributions

RB designed and conceptualized the study, collected and interpreted the data, and drafted the manuscript for intellectual content. AL designed and conceptualized the study, collected the data, ran the analyses and interpreted the data, and revised the manuscript for intellectual content. AN designed and conceptualized the study, interpreted the data, and revised the manuscript for intellectual content. MC, CEC, CD'A, LD, GL, and TL collected the data and revised the manuscript for intellectual content. MZ interpreted the data and revised the manuscript for intellectual content. RM designed and conceptualized the study, collected the data, ran the analyses and interpreted the data, and drafted and revised the manuscript for intellectual content. All authors approved the final manuscript.

## Conflict of Interest

The authors declare that the research was conducted in the absence of any commercial or financial relationships that could be construed as a potential conflict of interest.

## Footnotes

^1^Available online at: https://www.ecdc.europa.eu/en/geographical-distribution-2019-ncov-cases (accessed July 27, 2020).
