# Supplementary material for: Changes in Motor, Cognitive, and Behavioral Symptoms in Parkinson's Disease and Mild Cognitive Impairment During the COVID-19 Lockdown
Source: Front Psychiatry. 2020 Dec 14;11:590134. doi: 10.3389/fpsyt.2020.590134 (PMC7768013; doi:10.3389/fpsyt.2020.590134)
Supplement: Supplementary file 1 [file Data_Sheet_1.docx]

**Supplementary Table 1a. Changes in Neuropsychiatric Inventory during COVID-19 lockdown in PD-NC e PD-MCI**

|  | **PD-NC**  **(n=34)** | **PD-MCI**  **(n=31)** | **p-value** |
| --- | --- | --- | --- |
| Delusions | 0 | 2 (6.4) | 0.132 |
| Hallucinations | 2 (5.9) | 4 (12.9) | 0.329 |
| Agitation/Aggression | 0 | 1 (3.2) | 0.291 |
| Depression/Dysphoria | 9 (26.5) | 15 (48.4) | 0.067 |
| Anxiety | 12 (35.3) | 13 (41.9) | 0.583 |
| Elation/Euphoria | 0 | 3 (9.7) | 0.063 |
| Apathy/Indifference | 5 (14.7) | 9 (29.0) | 0.161 |
| Disinhibition | 0 | 2 (6.5) | 0.132 |
| Irritability/Lability | 7 (20.6) | 8 (25.8) | 0.618 |
| Aberrant Motor Behavior | 1 (2.9) | 5 (16.1) | 0.067 |
| Nighttime Behaviors | 11 (32.4) | 15 (48.4) | 0.188 |
| Appetite/Eating | 8 (23.5) | 5 (16.1) | 0.456 |

PD-NC, Parkinson’s Disease with Normal Cognition; PD-MCI, Parkinson’s Disease with Mild Cognitive Impairment

**Supplementary Table 1b. Changes in MDS-UPDRS Part I and Part II during COVID-19 lockdown in PD-NC and PD-MCI**

|  | PD-NC (n=34) | PD-MCI (n=31) | p-value |
| --- | --- | --- | --- |
| *MDS-UPDRS, Part I (Yes, %)* |  |  |  |
| Cognitive impairment | 7 (20.6) | 14 (45.2) | **0.034** |
| Hallucinations, psychosis | 2 (5.9) | 4 (12.9) | 0.329 |
| Depressed mood | 9 (26.5) | 15 (48.4) | 0.067 |
| Anxious mood | 12 (35.3) | 13 (41.9) | 0.583 |
| Apathy | 5 (14.7) | 9 (29.0) | 0.161 |
| Features of DDS | 1 (2.9) | 2 (6.5) | 0.500 |
| Sleep problems | 7 (20.6) | 13 (41.9) | 0.063 |
| Daytime sleepiness | 6 (17.6) | 11 (35.5) | 0.102 |
| Pain and other sensations | 8 (23.5) | 12 (38.7) | 0.185 |
| Urinary problems | 1 (2.9) | 4 (12.9) | 0.132 |
| Constipation problems | 4 (11.8) | 4 (12.9) | 0.889 |
| Light headedness on standing | 2 (5.9) | 3 (9.7) | 0.566 |
| Fatigue | 8 (23.5) | 15 (48.4) | **0.036** |
| *MDS-UPDRS, Part II (Yes, %)* |  |  |  |
| Speech | 2 (5.9) | 9 (29) | **0.013** |
| Saliva and Drooling | 3 (8.8) | 4 (12.9) | 0.596 |
| Chewing and swallowing | 2 (5.9) | 4 (12.9) | 0.329 |
| Eating tasks | 3 (8.8) | 4 (12.9) | 0.596 |
| Dressing | 2 (5.9) | 5 (16.1) | 0.183 |
| Hygiene | 3 (8.8) | 5 (16.1) | 0.371 |
| Handwriting | 5 (14.7) | 3 (9.7) | 0.538 |
| Hobbies and other activities | 3 (8.8) | 2 (6.5) | 0.720 |
| Turning in bed | 8 (23.5) | 9 (29.0) | 0.614 |
| Tremor | 12 (35.3) | 8 (25.8) | 0.408 |
| Getting out of bed | 6 (17.6) | 9 (29.0) | 0.277 |
| Walking and balance | 10 (29.4) | 10 (32.3) | 0.804 |
| Freezing | 6 (17.6) | 2 (6.4) | 0.170 |

PD-NC, Parkinson’s Disease with Normal Cognition; PD-MCI, Parkinson’s Disease with Mild Cognitive Impairment; MDS-UPDRS, Movement Disorder Society Unified Parkinson’s Disease Rating Scale; DDS, Dopamine Dysregulation Syndrome

**Supplementary Table 2a. Changes in Neuropsychiatric Inventory during COVID-19 lockdown in PD-MCI and MCInoPD**

|  | **PD-MCI**  **(n=31)** | **MCInoPD**  **(n=31)** | **p-value** |
| --- | --- | --- | --- |
| Delusions | 2 (6.5) | 1 (3.2) | 0.554 |
| Hallucinations | 4 (12.9) | 1 (3.2) | 0.162 |
| Agitation/Aggression | 1 (3.2) | 0 | 0.313 |
| Depression/Dysphoria | 15 (48.4) | 8 (25.8) | 0.066 |
| Anxiety | 13 (41.9) | 8 (25.8) | 0.180 |
| Elation/Euphoria | 3 (9.7) | 1 (3.2) | 0.301 |
| Apathy/Indifference | 9 (29.0) | 6 (19.4) | 0.374 |
| Disinhibition | 2 (6.5) | 0 | 0.151 |
| Irritability/Lability | 8 (25.8) | 7 (22.6) | 0.767 |
| Aberrant Motor Behavior | 5 (16.1) | 1 (3.2) | 0.086 |
| Nighttime Behaviors | 15 (48.4) | 5 (16.1) | **0.007** |
| Appetite/Eating | 5 (16.1) | 4 (12.9) | 0.718 |

PD-MCI, Parkinson’s Disease with Mild Cognitive Impairment; MCInoPD, Mild Cognitive Impairment not associated with Parkinson’s Disease

**Supplementary Table 2b. Changes in MDS-UPDRS Part I and Part II during COVID-19 lockdown in PD-MCI and MCInoPD**

|  | PD-MCI (n=31) | MCInoPD (n=31) | p-value |
| --- | --- | --- | --- |
| *MDS-UPDRS, Part I (Yes, %)* |  |  |  |
| Cognitive impairment | 14 (45.2) | 11 (35.5) | 0.437 |
| Hallucinations, psychosis | 4 (12.9) | 1 (3.2) | 0.162 |
| Depressed mood | 15 (48.4) | 8 (25.8) | 0.066 |
| Anxious mood | 13 (41.9) | 8 (25.8) | 0.180 |
| Apathy | 9 (29.0) | 6 (19.3) | 0.374 |
| Features of DDS | 2 (6.5) | 0 | 0.151 |
| Sleep problems | 13 (41.9) | 5 (16.1) | **0.025** |
| Daytime sleepiness | 11 (35.5) | 5 (16.1) | 0.082 |
| Pain and other sensations | 12 (38.7) | 1 (3.2) | **0.001** |
| Urinary problems | 4 (12.9) | 0 | **0.039** |
| Constipation problems | 4 (12.9) | 0 | **0.039** |
| Light headedness on standing | 3 (9.7) | 0 | 0.076 |
| Fatigue | 15 (48.4) | 6 (19.4) | **0.016** |
| *MDS-UPDRS, Part II (Yes, %)* |  |  |  |
| Speech | 9 (29.0) | 2 (6.5) | **0.020** |
| Saliva and Drooling | 4 (12.9) | 0 | **0.039** |
| Chewing and swallowing | 4 (12.9) | 2 (6.5) | 0.390 |
| Eating tasks | 4 (12.9) | 2 (6.5) | 0.390 |
| Dressing | 5 (16.1) | 1 (3.2) | 0.086 |
| Hygiene | 5 (16.1) | 1 (3.2) | 0.086 |
| Handwriting | 3 (9.7) | 1 (3.2) | 0.301 |
| Hobbies and other activities | 2 (6.5) | 1 (3.2) | 0.554 |
| Turning in bed | 9 (29.0) | 1 (3.2) | **0.006** |
| Tremor | 8 (25.8) | 1 (3.2) | **0.012** |
| Getting out of bed | 9 (29.0) | 0 | **0.001** |
| Walking and balance | 10(32.3) | 1 (3.2) | **0.003** |
| Freezing | 2 (6.5) | 0 | 0.151 |

PD-MCI, Parkinson’s Disease with Mild Cognitive Impairment; MCInoPD, Mild Cognitive Impairment not associated with Parkinson’s Disease; MDS-UPDRS, Movement Disorder Society Unified Parkinson’s Disease Rating Scale; DDS, Dopamine Dysregulation Syndrome
